# Medical Literature in the United States

**Published:** 1848-07

**Authors:** 


### MEDICAL LITERATURE IN THE UNITED STATES.

BY A MEMBER OF THE NATIONAL MEDICAL ASSOCIATION.

In the following sketch, which is mainly, as regards books, a classified catalogue, no critical examination has been attempted. The reasons soon to be assigned, when speaking of the Journals, for the difficulty of successful criticism in them, are deemed to be applicable on the present occasion. The mere enumeration, however, of the works on the different branches of medicine and the collateral sciences, published within the last five years, and rarely does our retrospection extend beyond this period, will, it is hoped, be gratifying to nearly all our professional brethren. They will see in it the copiousness of our medical literature, and be enabled, in consequence, to make selections in the purchase and perusal of books, the titles of which were previously unknown to many of them. Some, on seeing the present list, may feel ashamed at the meagre exhibition on their shelves, and be desirous of procuring one, at least, of the numerous works on each of the class of subjects brought under their notice.

### PERIODICAL LITERATURE.

The Medical Journals now edited and published in the United States are seventeen in number, independently of the American Journal of Pharmacy, and the American Journal and Library of Dental Science. The reprints of foreign periodicals are, the British and Foreign Medico-Chirurgical Review, and the Lancet, Braithwaite's Retrospect and Ranking's Abstract.

The American Journals exhibit nearly the same general arrangement of their contents and community of features. The three-fold division of their contents is into, 1st. Original communications—essays, history of diseases, accounts of cases—hospital or in private practice; and sometimes the medical topography of a district of country in connexion with a description of its endemic diseases. 2d. Reviews and bibliographical notices of recent publications. 3d. A summary of facts and discoveries in the several departments of medicine. The *staple* for the first division, especially in the Journals of the South and West, consists of descriptions of and disquisitions into the pathology and treatment of periodical fevers —intermittent and remittent—with their congestive complications, and of yellow fever. Of late years, the variety of continued fever, termed typhoid, has engaged the attention of physicians and medical writers throughout the country more than heretofore. Epidemic erysipelas, under various popular names, has also been described with considerable fulness in the journals in different sections of the Union. Within a still shorter period, meningitis, or cerebro-spinal arachnitis, as it is less accurately called, prevailing epidemically in some parts of the United States, as it had done previously in Europe, and particularly France, has become a subject for description in our journals.

Details of surgical operations, and particularly those for the removal of deformities, occupy a prominent place in the original department. Great boldness, seconded by considerable ingenuity, and some attention to the pathology and recuperative processes of the tissues, have made this branch of surgery a favourite one in the United States—taking our periodical medical literature as an exponent.

Although a very severe logic does not govern, in general, the literature of our medical journals, yet it is evident that greater stress now than formerly is laid on statistical estimates, or the numeral method, as it is sometimes termed, in both medical and surgical clinics, and in both pathological and therapeutical investigations.

The review department of our medical journals is not remarkable for vigour and comprehensiveness. Want of time for a careful perusal of the work, and a study of the entire subject; want of room for an adequate development of its features, scope and merits; and restraints, personal and conventional, on the free expression of opinion, are so many causes interfering with the satisfactory discharge of the duty of the reviewer. But, although a thorough critical review be thus prevented from appearing, the same obstacles do not exclude a regular analytical review, which might give the readers of a journal a tolerably adequate idea of the range of description or inquiry, and the general purposes of the author. Bibliographical notices ought always to possess this merit. That there is the ability to come up to the higher standard of reviewing, almost all our journals, from time to time, give evidence, when the reviewer meets with a favourite subject in the work before him.

Pertaining to the periodical literature of medicine, although not coming under the head of journals, are the Annual Reports of the Physicians of the numerous Asylums for the Insane in the United States, and of the medical attendants on Penitentiaries and other prisons. A large body of facts and suggestions, contributing to a system of medical Psychology, is contained in these reports. Their literary character and scientific value would be increased by an uniform method of statistical statement and deduction. Of the growing richness of this almost new branch of medical literature—the construction and best arrangement of the apartments of a lunatic asylum, the treatment, hygienic, medical and moral, of the insane—evidence has been furnished within the last few years, in the establishing and successfully sustaining a journal devoted entirely to this subject, called the "Journal of Insanity."

In the department of Dental Surgery there is considerable literary activity, manifested in the publication of periodicals devoted exclusively to the consideration of the theory and practice of this art, and to imparting the desirable qualifications, both scientific and ethical, to those who devote themselves exclusively to it. Prominent, by its acknowledged character and duration, among these, is the "American Journal and Library of Dental Science."

Transactions of State Medical Societies, as those of New York and Tennessee, embracing an account of the medical topography and diseases of different counties, and papers on particular diseases, or questions of pathology and therapeutics, constitute valuable additions to the periodical literature of medicine. Akin to these are the Transactions of Medical Associations, such as the Summary of those of the College of Physicians of Philadelphia, in which we read annual reports on the different branches of medicine, and of the conversations of the members on the prevalent diseases, and on any peculiarities in their features or treatment.

Under the head of periodical medical literature should be placed the Commemorative Addresses, in the form of Introductories and Valedictories, delivered by the Professors at the beginning and termination of the courses of instruction in the several Medical Schools.

### WORKS, IN BOOK FORM, ON MEDICINE AND ITS COLLATERAL BRANCHES.

The publications, in book form, within the past year, evince continued activity of the medical mind, and large contributions to our medical literature. A claim to originality can hardly be advanced in favour of our most instructive writers generally. Medicine seldom admits of this kind of display. Like history, it must consist mainly of a carefully digested summary of previously known and recorded facts, enforced with more or less freshness or vivacity of remark and commentary. A solitary discovery, and few can hope for more, will not constitute the institutes of medicine, nor can the most pregnant suggestions of a writer or teacher be expanded into a volume, or a course of lectures.

The chief productions of our home literature are systematic works on medicine and its several branches; and if we can form an opinion from the increasing demand for these, we should augur favourably for the curiosity of the great body of our medical men in the United States, which, in seeking, first, for the chief outlines of science, impels soon to a desire for acquaintance with its component parts and details. As works of great labour, embracing a vast amount of matter, and arranged for the purpose of ready reference and easy comprehension, the different systematic treatises on the *Theory and Practice of Medicine* are entitled to favourable appreciation. Of these, there have appeared during the past year the Treatises by Dr. Wood, of the University of Pennsylvania, and Dr. Dunglison, of the Jefferson Medical College; also, Lectures by Drs. Bell & Stokes. Each of these three works is in two volumes; the two last mentioned are new editions. The propriety of introducing the Lectures just mentioned, in this place, will be admitted, when it is known that the proportion of the whole work written by Dr. Bell is four-fifths, or about fourteen hundred pages. A new edition of the Lectures of Dr. Watson, of London, on the Practice of Physic, with additions by Dr. Condie, has also appeared within the year. The mention of these works suggests a reference to Dr. Dickson's Essays on Pathology and Therapeutics, in two volumes; to Mackintosh's Practice, edited by Dr. S. G. Morton; and Elliotson's, by Dr. Stewardson; which, although not published within the year, are of comparatively recent production. Under the same category will come the two volumes of published Lectures on Diseases of the Thoracic and Abdominal Viscera; and on Eruptive Fevers, Gout, Dropsy, &c., by Dr. Chapman. Also, a Compendium of Dr. Chapman's Lectures, by Dr. Benedict. Nor should we omit to mention the Cyclopædia of Medicine, edited by Dr. Dunglison; and Copland's Dictionary of Practical Medicine, still in progress of publication, edited by Dr. Lee, of the Geneva Medical College.

Under the head of systematic works on parts of medicine proper, which have been published within the year, are the Treatises on the Fevers of the United States, by Dr. Bartlett, of the Transylvania University, and by Dr. Alfred Stillé, on General Pathology. Shortly before this period was issued the Treatise on Fevers, by Dr. Clymer.

*Materia Medica*, within the above period, has found exponents in new editions of the Dispensatory of the United States, by Drs. Wood & Bache, and of the work on Materia Medica and Therapeutics, by Dr. Eberle. Very little retrospection displays to us the kindred works of Dr. John P. Harrison, of the Ohio Medical College; and of Dr. Dunglison.

In *Medical Botany* we have the works of Dr. R. E. Griffith, and of Dr. Carson, of the Philadelphia College of Pharmacy: the first is illustrated by numerous wood-engravings; the latter consists of 100 beautifully coloured plates, of the natural size of the plants, with letter-press explanations.

Republications of Dr. Pereira, of Ballard and Garrod, and of Royle on Materia Medica and Therapeutics, with additions by American editors, (Dr. Carson for the first and third, and Dr. Griffith for the second of these works,) have been made within a late period. Mayne's Dispensatory, edited by Dr. Griffith, is just issued.

In the department of *Surgery* we meet with Dr. Sargeant's

"Minor Surgery," which had been preceded, not long before, by Dr. Henry H. Smith's volume on the same subject. Of higher aim and wider range is the treatise on the Principles and Practice of Surgery, by Dr. Geo. McClellan, edited by his son, Dr. J. B. McClellan. The large work of Dr. Pancoast, of the Jefferson Medical College, on Operative Surgery, was published but a few years ago, and has since then reached a second edition. Of a mixed character, in its being translated, and at the same time in its having received large additions from the pens of its American translator and editor, Dr. Townsend, and of Dr. Mott, is the great work of Velpeau on Operative Surgery. The System of Chelius, just issued from the press in this country, is another monument of laborious investigation in this department. The republication of elementary treatises on surgery has been very active of late years, as is evident by reference to Liston's Elements, and Practical and Operative Surgery, which have been edited, severally, by Dr. Gross, of the Louisville University, Dr. Norris, of the Pennsylvania Hospital, and Dr. Mütter, of the Jefferson Medical College; also, to the Principles and Practice of Surgery, by Mr. Druitt, edited by Dr. Flint; Ferguson's Practical Surgery, edited by Dr. Norris; and to the Principles of Surgery, and the Practice of Surgery, two separate works, by Mr. Miller, of the University of Edinburgh; and Samuel Cooper's First Lines, edited by Dr. Parker, of the College of Physicians and Surgeons, New York. Nor must we omit to notice, in this connexion, as having been republished within a few years, Samuel Cooper's Surgical Dictionary, edited by Dr. Reese, of New York; Sir Astley Cooper's Entire Surgical Works, in four volumes; and quite recently, the several treatises and lectures of Sir Benjamin Brodie.

Within the year, *Ophthalmic Surgery* has been represented by the republished Treatises of Mr. Lawrence, and of Mr. T. Wharton Jones, with additions to both by Dr. Hays. At a somewhat earlier date appeared the Manual of Diseases of the Eye, by Dr. Littell.

*Midwifery* has been represented during the past year by the Manual of Obstetrics of Dr. Tucker, of the Franklin Medical College; by the republication of Spratt's Obstetric Tables; and of new editions of Churchill and of Ramsbotham. Churchill is under the editorial supervision of Dr. Huston, of the Jefferson Medical College. The peculiarity of Spratt's Tables consists in the pictorial illustrations being representations of successive layers of tissue and teguments, by means of "dissected plates," which may be raised in succession, so as to exhibit the parts beneath. Within a comparatively brief period, there has been quite an outpouring from the press of volumes on Obstetrics. Witness the systematic works of Hamilton, Collins, Rigby, Velpeau, (the last translated by Dr. C. D. Meigs, of the Jefferson Medical College, and edited by Dr. Wm. Harris,) Lee, Moreau, Chailly, and Murphy, all republished in this country. Moreau was translated by Dr. Betton, of the Franklin Medical College; and Chailly by Dr. Bedford, of the University of the City of New York, who also made annotations on the original text. To these we must add Maygrier's Illustrated Midwifery translated by Dr. Doane.

On *Diseases of Females* there have lately appeared the volume of "Letters to his Class," by Dr. C. D. Meigs; and a republication of a new edition of Churchill, with additions by Dr. Huston; also of Whitehead on Abortion and Sterility. Not long before, the elaborate Treatise of Columbat was translated and enriched with numerous additions by Dr. Meigs; and that of Ashwell, edited by Dr. Goddard, of the Franklin Medical College. The valuable Treatise of Dr. Dewees is still sought for, and the demand is met by the issue of new editions.

For a long period the only work, in English, of any repute and currency, on *Diseases of Children*, was that of Underwood. His successor in usefulness and reputation in this department of medical literature was Dr. Dewees, whose Treatise soon became a work of authority and reference, for practical purposes, on both sides of the Atlantic. Since its first appearance, a little more than a quarter of a century ago, the works on Diseases of Children have greatly increased in number and value. Restricting ourselves to a notice of those of home authorship, or which have been republished in the United States, we meet, first, with the Treatise of Dr. Eberle on the Diseases and Physical Education of Children, which has gone through successive editions, the last within the year. If not actually coming within this period, yet of quite recent publication, is the second edition of Dr. Condie's Treatise. He had previously edited a second American edition of Drs. Evans and Maunsell's work on the same subject. The latter had been brought out in Bell's *Select Medical* Library, as was subsequently a new edition of Underwood, and also Dr. Colley's Treatise, to the former of which Dr. Bell added copious notes. The translation of Billard's Treatise, by Dr. Stewart, was followed, after a few years, by a work from the pen of Dr. Stewart himself, which, ere long, reached a second edition.

In *Anatomy*, the republications of Solly on the Human Brain, and of Von Behr's Hand-book of Human Anatomy, during the past twelvemonth, have been preceded by the issue of a new edition of Dr. Horner's Special Anatomy and Histology, and of the systematic treatise of Cruveilhier, edited by Dr. Pattison, of the University of the City of New York. Under this head come, also, Wilson's Anatomy, edited by Dr. Goddard; Wistar's, edited by Dr. Pancoast; Dr. Horner's United States Dissector, edited by Dr. Henry H. Smith; the Dublin Dissector, edited by Dr. Watts, of the New York College of Physicians and Surgeons; Wilson's Dissector, edited by Dr. Goddard, &c. Most of these are illustrated by wood-cuts. Important aids to the study of anatomy, which merit notice in the literature of the subject, have been brought out of late years, in the form of engravings, plain and coloured, of the several parts of the human frame. Among these we may mention the Anatomical Atlas by Dr. Henry H. Smith, in one volume, royal octavo; the Anatomical Plates, by Quain and Wilson, in large quarto form; and those of the Bloodvessels and Nerves, by Dr. Neil; and of the Heart and Arteries, by Dr. J. M. Allen.

*Pathological Anatomy* has not furnished any volume within the year; but if we go back for a short period we find the large and comprehensive work of Dr. Gross, and the republication of the Treatises of Hope, under the editorial care of Dr. Lawson, of the Ohio Medical College; and also those of Vogel and of Hasse. Dr. Gross's "Elements" is illustrated with numerous coloured engravings. Hope's "Principles and Illustrations" present a series of plates, coloured after the morbid states of the organs. Vogel's Treatise, containing also numerous engravings, is on General Pathological Anatomy, and is introductory to a second part, which will exhibit an account of the morbid changes affecting particular organs. Hasse's volume is on the Morbid Anatomy of the Organs of Circulation and of Respiration.

In *Physiology* we have to announce the republication of Dr.

Carpenter's Principles, being the third American edition, which, like the two preceding ones, has been brought out under the editorial supervision of Dr. Clymer. Of recent issue is a new edition of Dr. Dunglison's Human Physiology; and not long antecedently appeared an Abridgement of Müller's Elements, by Dr. Bell; and a new edition of Magendie's Treatise, edited by Dr. Revere. Todd & Bowman's Anatomical Physiology and Physiology of Man is in course of republication. The smaller volumes by Dr. Reece, Dr. Jarvis, and Dr. Reynell Coates, are well adapted to medical students in their early period of study, and to collegiate and school instruction. The "Essay towards a Correct Theory of the Nervous System," by Dr. John Harrison, of the Louisiana Medical College, is an important contribution to Physiology.

The recent republication of Wilson on *Diseases of the Skin* is farther evidence of the improved literature of this branch of medicine, or *Dermatology*, as it is sometimes called. Three years ago appeared, in an American dress, the large and classical work of Rayer, illustrated with all the numerous coloured plates of the original French, and edited by Doctor Bell. Nearly contemporaneous was the Synopsis, by Dr. Worcester, of the Medical School of Cleveland, with "sixty coloured figures;" and the Manual of Cazenave & Schedel, edited by Dr. Bulkley, of New York, Lecturer on Diseases of the Skin. The Treatise of Plumbe, and the more restricted Observations of Green & Dendy were republished some years antecedently, the first in Dr. Bell's, the last two in Dr. Dunglison's "Library."

The *Manual* of Dr. Taylor on *Toxicology*, edited by Dr. R. E. Griffith, which has been republished within the twelvemonth, suggests a notice of that of Christison on Poisons, which was brought out in Bell's Medical Library. It is but recently since American medical and legal readers have enjoyed the advantages of ready reference to systematic works on Medical Jurisprudence, in addition to the long and widely known and esteemed Treatise of the Doctors Beck. In the same year, [1845] were republished "Medical Jurisprudence," by Dr. Taylor, edited by Dr. R. E. Griffith, and Dr. Guy's "Principles of Forensic Medicine," edited by Dr. Lee. The "Medical Jurisprudence of Insanity," by Dr. Ray, of Maine, has reached a second edition.

In the science of *Chemistry*, with which medicine has collaterally such frequent relations, we see no work brought out among us during the last year. But a little anterior to this time, we meet with the systems of Graham, Kane, and Turner, the first edited by Dr. Bridges, of the Franklin Medical College, the second by Dr. Draper, of the University of the City of New York, and the last by Dr. James B. Rogers of the Pennsylvania University, and by Dr. Robert E. Rogers of the Virginia University. This last volume includes Gregory's Outlines of Organic Chemistry. Of still more direct application to physiology and pathology are the Treatises on Animal Chemistry by Liebig, and by Simon, both of which have been republished in the United States. Resting on a similar basis, but with more diversified Therapeutical deductions and advice, are the Treatise by Prout on "Stomach and Renal Diseases," and that by Budd on "Urinary Deposits;" also republications. The smaller volumes on General Chemistry, of Fownes, of Hoblyn, and of Johnston, of the Wesleyan University, Connecticut, the last on the basis of Turner, are useful for students.

In *Natural Philosophy*, the collateral relations of which with medicine are, also, acknowledged, we find among the year's republications Bird's works, under this head, and that of Müller on Physics and Meteorology. Of still more direct application are Matteucci's "Lectures on the Physical Phenomena of Human Beings."

Under the head of *Hygiene* there has been a new edition of Dr. Combe's Treatise on the Management of Infancy, edited by Dr. Bell. Of anterior, but still comparatively recent date, are the Essay of Dr. John C. Warren on Physical Education and the Preservation of Health; the Elements of Hygiene, by Dr. Dunglison; and the Treatises of Dr. Bell on Regimen and Longevity, of Dr. Sweetser on Mental Hygiene, and of Dr. Brigham on Mental Excitement and Cultivation.

Among the elementary aids to Medical Studies, we may notice Dr. Mendenhall's Medical Student, Dr. Ludlow's Manual of Examinations, and Dr. Dunglison's Medical Student; all of which have reached second editions—also Hooper's Examinations. Of the same kind are the several *Dictionaries* by Drs. Dunglison and Gardner, and the republished one of Hoblyn, edited by Dr. Hays.

In special directions, medical literature has been rich among us of late years. On *Thoracic Diseases*, the work republished within the year, and on this account first to be noticed, is the Treatise of Blakiston on the Chest. Before this we had the works of Drs. Stokes, Gerhard, Andral and Williams on Thoracic Diseases; of Dr. Horace Green on Diseases of the Air Passages; and those of Hope and Latham, together with home translations of the manuals of Aran and Andry, on Diseases of the Heart. Aran was translated by Dr. William A. Harris of the U. S. Navy; Andry, by Dr. Kneeland, of Boston. On Physical Diagnosis of the Diseases of the Lungs we have the Manual of Walshe, and of the Lungs and Heart, the Manuals of Hughes, and of Barth and Roger. Of this last a translation has been made by Dr. Francis G. Smith, of Philadelphia, and another by Dr. Newbigging, of Edinburgh, to the second of which Dr. Lawson has added an Appendix.

But few monographs, not included in preceding divisions, have been published during the past year. We may mention Dr. Burrows on Cerebral Circulation, M. Lallemand on Spermatorrhœa, and Drs. Reese, Markwick and Griffith on Blood and Urine, republications within this period. Somewhat earlier we meet with Dr. Bartlett's Essay on the Philosophy of Medical Science, Budd on Diseases of the Liver, Phillips on Scrofula, Fordyce on Fever, Billings' First Principles of Medicine, Tamplin on Deformities, Curling on the Anatomy, Physiology and Diseases of the Testes, Walshe on Cancer, edited by Dr. John M. Warren, Esquirol on Insanity, translated by Dr. E. K. K. Hunt, Johnson and Martin on the Influence of Tropical Climates, &c., Adulteration of Various Substances, beside Medicine and the Arts, by Dr. Lewis C. Beck, Domestic Management of the Sick Room, by Dr. A. T. Thomson, edited by Dr. R. E. Griffith, Ricord on Venereal Diseases, and Lugol on Scrofulous Diseases, both translated by Dr. Doane, and Acton on Venereal Diseases.

The reference made to Dental Surgery in connexion with our periodical literature ought to be renewed now, when we are speaking of the publication of separate works, in volumes. In addition to the valuable treatises which have appeared at intervals in the American Library of Dental Science, mention should be made of the "Principles and Practice of Dental Surgery," by Dr. Chapin A. Harris, Fox's "Natural History and Diseases of the

Teeth," remodelled and largely added to by Dr. C. A. Harris, Maury's "Treatise on Dental Art," translated by J. B. Savier; and the handsome quarto of Dr. Goddard, on the "Anatomy, Physiology and Pathology of the Human Teeth," with the requisitely applied Surgery.

